# Lower serum insulin-like growth factor-1 levels are independently associated with anemia in patients undergoing maintenance hemodialysis

**DOI:** 10.1080/0886022X.2023.2221130

**Published:** 2023-07-05

**Authors:** Shilin Xu, Jun Ren, Yuping Yao, Danping Qin, Yan Liu, Xiaoshi Zhong, Rongshao Tan, Chunjie Jiang, Yun Liu, Wenxuan Chen

**Affiliations:** a Department of Nephrology, Guangzhou Red Cross Hospital of Jinan University, Guangdong Province, P.R. China; b Department of Nephrology, The 2nd People’s Hospital of Bijie, Bijie, Guizhou Province, P.R. China; c Guangzhou Institute of Disease-Oriented Nutritional Research, Guangzhou Red Cross Hospital of Jinan University, Guangzhou, Guangdong Province, P.R. China

**Keywords:** Serum insulin-like growth factor-1, anemia, hemoglobin, maintenance hemodialysis

## Abstract

The relationship between serum insulin-like growth factor-1 (IGF-1) levels and anemia in patients undergoing maintenance hemodialysis (MHD) remains unclear. This cross-sectional study included patients who underwent MHD treatment for >3 months at our dialysis center in March 2021. Demographic and clinical data were recorded. Blood samples were collected before the hemodialysis sessions, and general serum biochemical parameters, routine blood markers, and serum IGF-1 levels were measured. Patients were divided into a group without anemia (hemoglobin ≥110 g/L) and a group with anemia (hemoglobin <110 g/L), and multivariable linear and binary logistic regression analyses were performed to study the relationship between the levels of serum IGF-1 and anemia. A total of 165 patients (male/female = 99:66) with MHD were enrolled in the study, with a median age of 66.0 (58.0, 75.0) years and a median dialysis vintage of 27.0 (12.0, 55.0) months. The mean hemoglobin level was 96.38 ± 16.72 g/L, and 126 patients had anemia (76.4%). Compared to patients without anemia, patients with anemia had lower serum IGF-1 and triglyceride levels and higher intravenous iron supplementation on dialysis (all *p* < 0.05). After adjusting for confounding factors in different models, the nine-model multivariate binary logistic regression analyses also confirmed that lower serum IGF-1 levels and serum IGF-1 < 197.03 ng/ml were both independently associated with anemia in patients undergoing MHD. However, further multicenter studies with larger sample sizes are required to confirm these findings.

## Graphical Abstract

**Figure:**
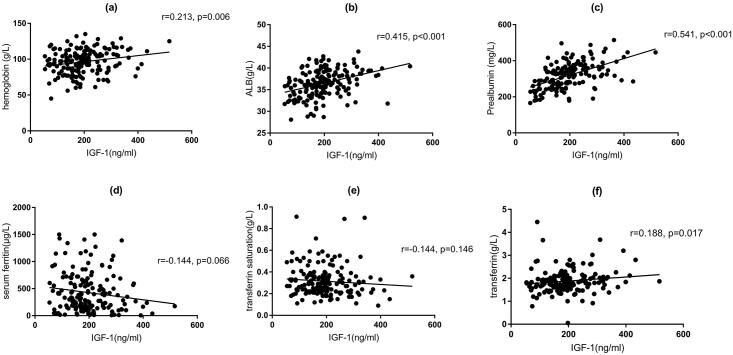

## Introduction

Anemia is a common complication in patients undergoing maintenance hemodialysis (MHD) and is associated with an increased risk of cardiovascular events and all-cause mortality [1]. In 2016, the Dialysis Outcomes and Practice Patterns Study in China reported that the proportion of patients undergoing MHD with hemoglobin levels less than 90 g/L was 21%, compared to 10% and 3% in North America and Japan, respectively [2]. Although iron and erythropoietin treatments have been widely used in recent years, the current situation still warrants greater attention and further improvements, and other factors contributing to anemia in patients undergoing MHD deserve to be explored.

Studies have shown that in nondiabetic adults [3] and older adult populations [4], higher levels of serum insulin-like growth factor (IGF-1) are independently associated with higher hemoglobin levels. It has also been reported that IGF-1 may play an important role in regulating erythropoiesis independent of erythropoietin levels in patients with erythrocytosis undergoing MHD [5]. However, few clinical studies have focused on the association between serum IGF-1 and hemoglobin levels in patients undergoing MHD. Thus, this cross-sectional study investigated the relationship between serum IGF-1 and hemoglobin levels in patients undergoing MHD.

## Materials and Methods

### Population

This single-center cross-sectional study included patients who underwent MHD at the Guangzhou Red Cross Hospital Hemodialysis Center in March 2021. The inclusion criteria were as follows: (1) dialysis vintage ≥3 months, three times a week, 4 h per visit; (2) age 18 years or older; and (3) informed consent. The exclusion criteria were as follows: (1) a history of continuous ambulatory peritoneal dialysis or renal transplantation before MHD; (2) a history of gastrointestinal bleeding or other acute blood loss within 6 months; (3) a history of blood transfusion within the past 3 months; (4) a history of trauma or surgery within the past 3 months; (5) severe infection, heart failure, or malignancy; and (6) refusal to participate in this study. This study was reviewed and approved by the Ethics Committee of Guangzhou Red Cross Hospital, Jinan University (ID: 2021–202-02).

### Demographic, clinical, and laboratory data

The demographic and clinical data of these patients were extracted from the data management system for hemodialysis (Hope®, software) developed by our group [6,7]. Venous blood samples were collected before the hemodialysis session and sent to the clinical laboratory at our hospital within 2 h. The laboratory parameters were as follows: Kt/v, serum insulin, serum IGF-1, blood urea nitrogen (BUN), serum creatinine, serum phosphorus, serum parathyroid hormone, serum vitamin B12, serum folic acid, serum high-density lipoprotein cholesterol, serum low-density lipoprotein cholesterol, serum total cholesterol, serum triglycerides, serum ferritin, serum transferrin, serum albumin, hemoglobin, serum prealbumin, serum interleukin-6, and serum high-sensitivity C-reactive protein. Drug prescriptions in the past 3 months were also recorded, including the dosages of recombinant human erythropoietin (rHuEPO) (U/kg/week) and iron supplementation (mg/kg/week).

Serum IGF-1 levels were measured by high-performance liquid chromatography–mass spectrometry (Thermo Q, Thermo Fisher, Massachusetts, US). According to the 2017 Kidney Disease: Improving Global Outcomes (KDIGO) guidelines [8], patients were divided into a group with anemia (hemoglobin < 110 g/L) and a group without anemia (hemoglobin ≥ 110 g/L).

The devices used for hemodialysis treatment were a Braun Dialog+ (B. Braun Co., Ltd., Melsungen, Germany) and a REXEED-15L high-throughput polysulfone membrane dialyzer (Asahi Kasei Corp., Tokyo, Japan) with a membrane area of 1.5 m^2^, dialysis blood flow rate of 200–300 mL/min, dialysis fluid flow rate of 500 mL/min, and dialysis duration of 4 h.

### Statistical methods

Descriptive statistics include the mean ± standard deviation for continuous variables with a normal distribution, the median (25–75% interquartile range) for data with a skewed distribution, and percentages for categorical variables. Differences between the two groups were tested using the *t* test, nonparametric test, or chi-square test, as appropriate. Pearson’s or Spearman’s analyses were used to explore the correlation between clinical parameters and serum IGF-1 levels. Univariate and multivariate binary logistic regression or multiple linear regression analyses were used to investigate the relationship between serum IGF-1 levels and anemia in patients undergoing MHD. Data were analyzed using SPSS (version 26.0; IBM Corp, Armonk, NY, USA) statistical software, and a *p* values <0.05 was considered to indicate statistical significance.

## Results

### Characteristics of the study population

**Figure 1.**
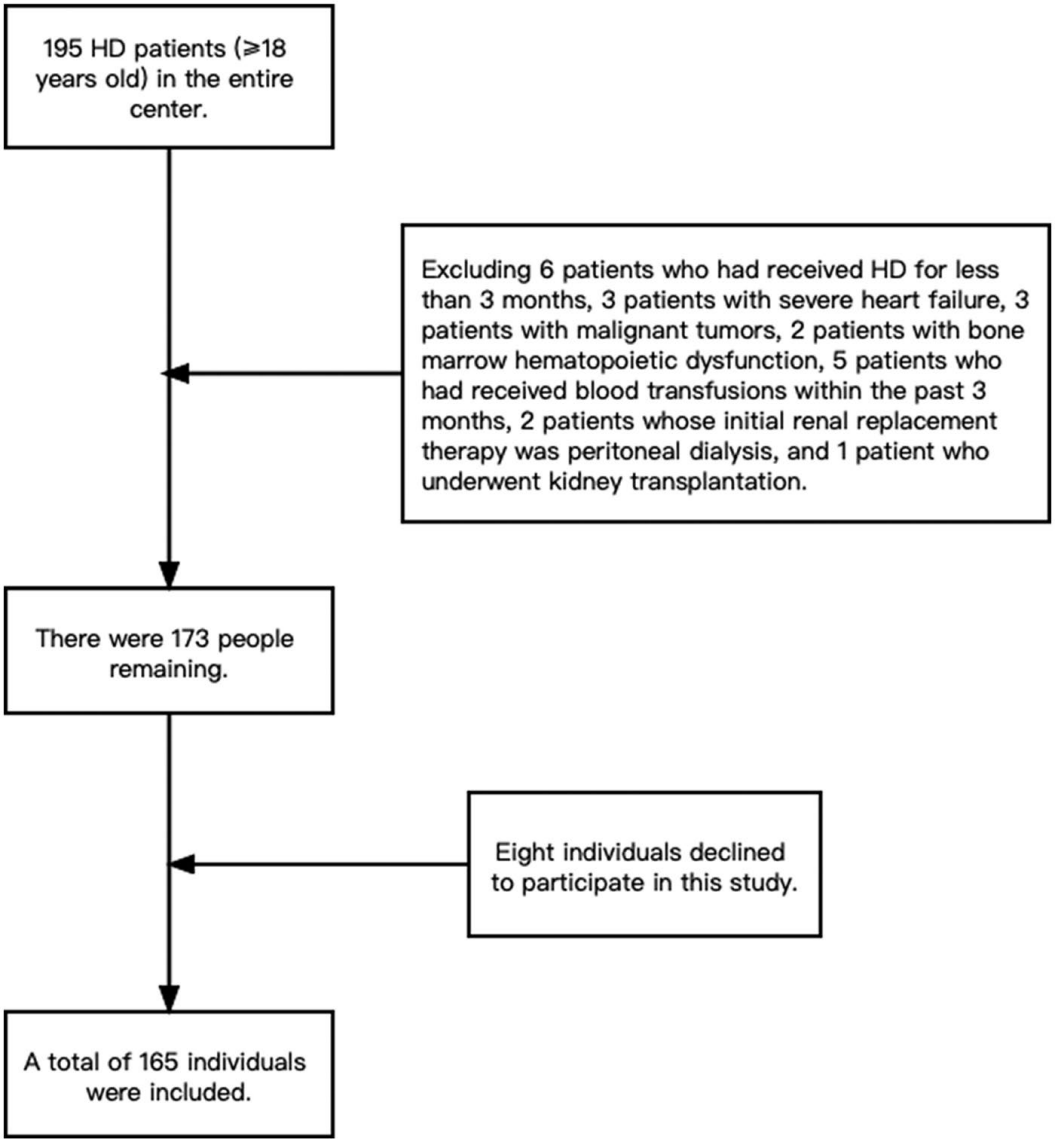
Flow chart for sample selection.

According to the inclusion and exclusion criteria, a total of 165 (male: female = 99:66) patients undergoing MHD were included in the study, with a median age of 66.0 (58.0, 75.0) years and a median dialysis vintage of 27.0 (12.0, 55.0) months, and 92 of them had diabetes (55.8%). The sample selection flowchart is presented in Figure 1. The mean hemoglobin level was 96.38 ± 16.72 g/L, and 126 (76.4%) patients had anemia.

### Comparison between groups

**Figure 2.**
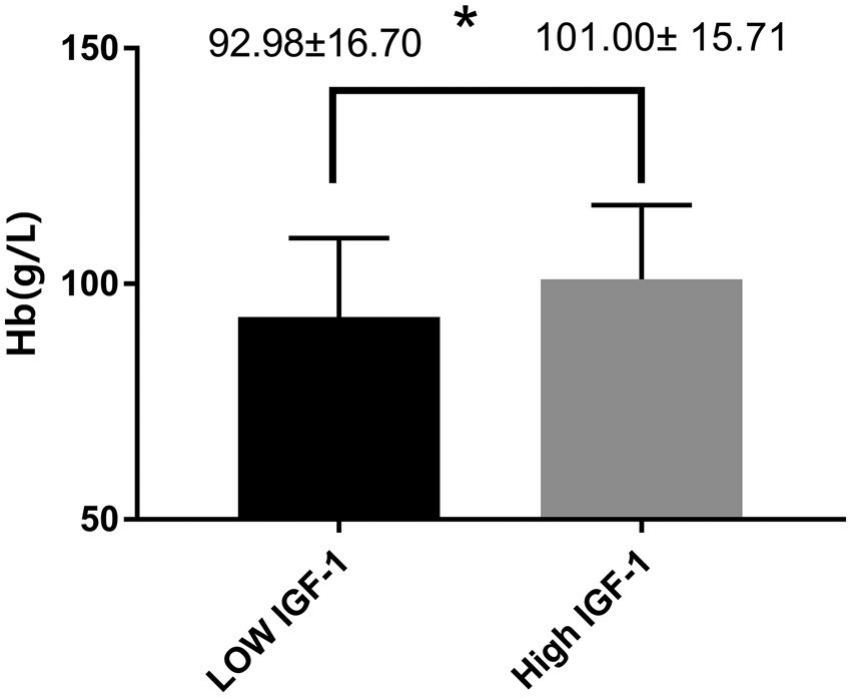
Comparison of hemoglobin levels between groups according to median serum IGF-1 in patients undergoing MHD. *p < 0.05. Hb: hemoglobin; IGF-1: insulin-like growth factor-1.

Compared with patients without anemia, patients with anemia had higher levels of intravenous iron supplementation (*p* = 0.047) and lower levels of serum IGF-1 (*p* = 0.007) and serum triglycerides (*p* = 0.008) (Table 1). Grouped by the mean serum IGF-1 level, the study participants were divided into two groups: a high IGF-1 group (IGF-1 ≥ 197.03 ng/ml) and a low IGF-1 group (IGF-1 < 197.03 ng/ml). Compared to the high IGF-1 group, the low IGF-1 group had a lower blood hemoglobin level (101.00 ± 15.71 vs. 92.98 ± 16.71 g/L, *p* = 0.002) (Figure 2).

**Table 1.** Clinical characteristics of the study population before and after stratification into groups by anemia.

Variables	Normal Ranges	Total (*n* = 165)	Patients without anemia (*n* = 39)	Patients with anemia (*n* = 126)	*p* value
Age (years)		65.79 ± 13.12	64.10 ± 13.42	66.31 ± 13.03	0.362
Sex, female (%)		66 (40.0)	20 (51.3)	46 (36.5)	0.145
DM, *n* (%)		92 (55.8)	24 (61.5)	68 (54)	0.517
Dialysis vintage (months)		38.31 ± 33.60	34.56 ± 29.83	39.48 ± 34.73	0.427
Kt/V		1.39 ± 0.47	1.35 ± 0.26	1.40 ± 0.52	0.565
Iron supplementation dosage (mg/kg/week)		1.72 (1.51, 1.93)	1.42 (1.34, 1.71)	1.7 (1.52, 1.94)	0.047
rHuEPO dosage (U/kg/week)		162.61 (137.03, 188.02)	162.3 (131.83, 199.02)	163.7 (139.21, 186.67)	0.541
Serum insulin (mIU/L)	3–25	16.12 (8.93, 24.71)	17.72 (9.46, 36.81)	15.0 (8.74, 24.32)	0.528
Serum IGF-1 (ng/mL)		197.03 ± 82.67	227.86 ± 98.43	187.49 ± 75.05	0.007
Blood urea nitrogen (mmol/L)	1.9–6.8	26.33 (23.42, 30.74)	27.32 (23.26, 30.17)	26.2 (23.44, 30.63)	0.833
Serum creatinine (μmol/L)	62–115	899.01(742.03, 1082.02)	961.01 (702.52, 1096.03)	896.01 (762.23, 1077.04)	0.763
Serum phosphorus (mmol/L)	1.00–1.60	2.22 ± 0.61	2.31 ± 0.75	2.12 ± 0.53	0.140
Serum parathyroid hormone (pmol/L)	1.6–6.9	30.61(16.91, 45.53)	26.9 (17.91, 46.42)	31.03 (16.51, 45.34)	0.732
Serum vitamin B12 (pg/mL)	187–883	307.8 (221.4, 432.6)	303.5 (249.0, 433.6)	309.2 (217.5, 428.3)	0.588
Serum folic acid (μg/L)	Folate deficiency can be diagnosed if the level is <5.9 (μg/L)	15.43 (11.02, 22.91)	14.02(10.41, 19.64)	16.12 (11.14, 23.42)	0.134
Serum high-density lipoprotein cholesterol (mmol/L)	1.10–2.00	1.11 (0.85, 1.43)	1.12 (0.91, 1.44)	1.11 (0.82, 1.43)	0.930
Serum low-density lipoprotein cholesterol (mmol/L)	1.90–3.30	2.43 (1.81, 2.90)	2.41 (2.01, 3.22)	2.32 (1.81, 2.98)	0.378
Serum total cholesterol (mmol/L)	3.9–5.2	4.32 (3.71, 5.01)	4.52 (3.92, 5.23)	4.31 (3.73, 5.02)	0.223
Serum triglycerides (mmol/L)	0.5–1.9	1.63 (1.12, 2.44)	2.0 (1.43, 3.01)	1.53 (1.04, 2.32)	0.008
Serum ferritin (μg/L)	23.9–336.2	324.81 (143.72, 617.53)	265.14 (159.62, 459.81)	358.93 (145.34, 690.05)	0.297
Serum transferrin (g/L)	2.00–3.60	1.81 (1.63, 2.04)	1.92 (1.61, 2.13)	1.81(1.64, 2.01)	0.323
Serum albumin (g/L)	34.0–53.0	36.52 ± 3.23	37.19 ± 2.91	36.30 ± 3.34	0.253
Serum prealbumin (mg/L)	200–450	326.01 (272.28, 362.87)	346.81 (281.22, 376.63)	321.91 (265.33, 358.01)	0.101
Serum interleukin-6 (pg/mL)	0–7.00	9.43 (6.21, 17.54)	9.51 (5.93, 16.64)	9.12 (6.33, 17.55)	0.865
Serum high-sensitive C-reactive protein (mg/L)	0–3.00	3.42 (1.61, 8.63)	3.01 (1.56, 9.71)	3.35 (1.63, 8.21)	0.875

DM: diabetes mellitus; rHuEPO: S recombinant human erythropoietin; IGF-1: insulin-like growth factor-1; Kt/v, urea clearance index used to quantify the dialysis treatment adequacy, where K is the dialyzer clearance of urea, t is the dialysis time, and V is the volume of distribution of urea approximately equal to the total body water of the patient.

### Associations between IGF-1 and anemia in patients undergoing MHD

**Figure 3.**
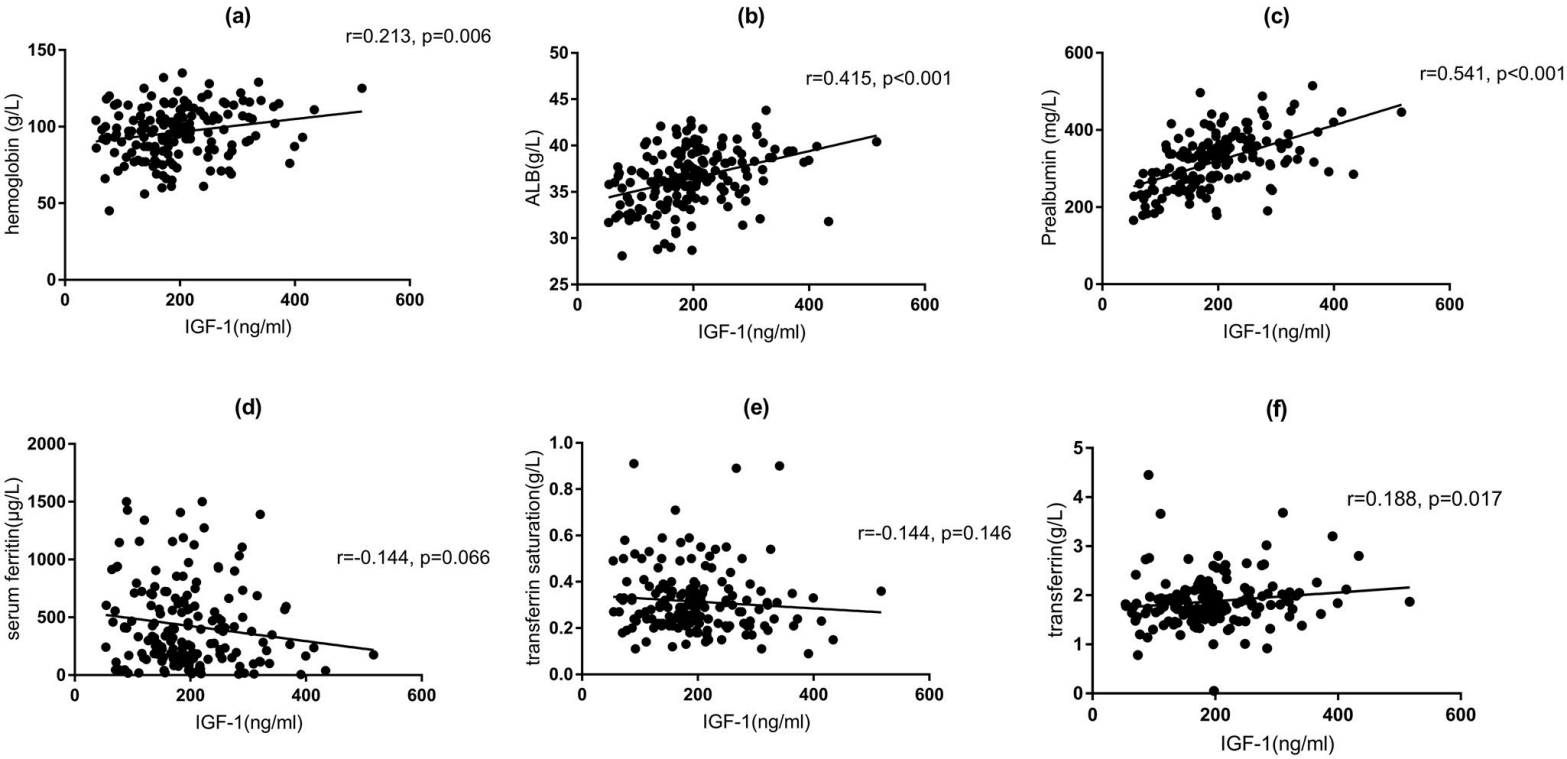
Correlation analysis between levels of hemoglobin, serum albumin, serum prealbumin, serum ferritin, transferrin saturation, serum transferrin, and levels of serum IGF-1. ALB: albumin; IGF-1: insulin-like growth factor-1.

In the correlation analysis of IGF-1 with clinical parameters, the results of Spearman’s analysis (Table 2) showed that IGF-1 was positively correlated with hemoglobin (*r* = 0.213, *p* = 0.006), serum albumin (*r* = 0.415, *p* < 0.001), serum prealbumin (*r* = 0.541, *p* < 0.001), and transferrin (*r* = 0.188, *p* = 0.017) (Figure 3).

**Table 2.** Correlation analysis of IGF-1 with clinical parameters.

Variables	*r* value	*p* value
Hemoglobin (g/L)	0.213	0.006
Age (years)	–0.505	<0.001
Dialysis vintage (months)	–0.028	0.725
Kt/V	–0.073	0.355
Iron supplementation dosage (mg/kg/week)	–0.028	0.726
rHuEPO dosage (U/kg/week)	–0.181	0.022
Serum insulin (mIU/L)	0.291	<0.001
Serum creatinine (μmol/L)	0.511	<0.001
Blood urea nitrogen	0.273	<0.001
Serum phosphorus (mmol/L)	0.333	<0.001
Serum parathyroid hormone (pmol/L)	0.194	0.013
Serum vitamin B12 (pmol/L)	–0.094	0.234
Serum folic acid (μg/L)	–0.070	0.383
Serum high-density lipoprotein cholesterol (mmol/L)	–0.234	0.003
Serum low-density lipoprotein cholesterol (mmol/L)	0.071	0.370
Serum very low-density lipoprotein cholesterol (mmol/L)	0.186	0.018
Serum total cholesterol (mmol/L)	0.027	0.731
Serum triglycerides (mmol/L)	0.259	<0.001
Serum ferritin (μg/L)	–0.144	0.066
Serum transferrin (g/L)	0.188	0.017
Serum albumin (g/L)	0.415	<0.001
Serum prealbumin (mg/L)	0.541	<0.001
Serum interleukin-6 (pg/mL)	–0.266	<0.001
Serum high-sensitivity C-reactive protein (mg/L)	–0.138	0.081

The results of the univariate binary logistic regression analyses are shown in Table 3. We observed that lower levels of serum IGF-1 (odds ratio [OR] = 0.99, 95% confidence interval [CI] = 0.99–1.00, *p* = 0.010), serum IGF-1 < 197.03 ng/mL (OR = 0.36, 95% CI = 0.17–0.75) *p* = 0.007), and serum triglycerides (OR = 0.77, 95% CI = 0.59–0.99, *p* = 0.044) (Table 3) were related to anemia. After adjustment for age, sex, dialysis vintage, diabetes, serum high-sensitivity C-reactive protein, iron supplementation dosage, rHuEPO dosage, serum triglycerides, serum albumin and prealbumin, the nine models’ multiple linear regression analyses showed that without the inclusion of serum albumin or prealbumin, higher levels of serum IGF-1 and serum IGF-1 ≥ 197.03 ng/ml were independently associated with higher hemoglobin levels. However, when serum albumin or prealbumin was included in the model, only serum IGF-1 ≥ 197.03 ng/ml was independently associated with higher hemoglobin levels (Table 4). After adjusting for the same confounders mentioned above in different models, the nine-model multivariate binary logistic regression analyses also confirmed that lower serum IGF-1 levels and serum IGF-1 < 197.03 ng/ml were both independently associated with anemia in patients undergoing MHD (Table 5).

**Table 3.** Univariate binary logistic regression between clinical parameters and anemia.

Variable	OR (95% CI)	*p*_value
Age (years)	1.012 (0.991, 1.043)	0.359
Sex: Female vs. Male	1.832 (0.891, 3.781)	0.102
DM	0.733 (0.352, 1.531)	0.406
Dialysis vintage (months)	1.012 (0.997, 1.021)	0.425
Serum high-sensitivity C-reactive protein (mg/L)	1.032 (0.981, 1.071)	0.257
Serum interleukin 6 (pg/mL)	1.012 (0.973, 1.044)	0.653
Serum albumin (g/L)	0.932 (0.833, 1.052)	0.252
Serum prealbumin (mg/L)	1.011 (0.992, 1.032)	0.207
Serum ferritin (µg/L)	1.022 (0.998, 1.044)	0.218
Serum transferrin (g/L)	0.592 (0.333, 1.171)	0.133
Iron saturation	2.412 (0.152, 37.933)	0.531
rHuEPO dosage (U/kg/week)	0.231 (0.992, 1.023)	0.655
Iron supplementation dosage (mg/kg/week)	3.373 (0.892, 12.692)	0.073
Serum IGF-1 (ng/mL)	0.993 (0.991, 1.022)	0.010
Serum IGF-1 ≥ 197.03 (ng/mL)	0.36 (0.17-0.75)	0.007
Kt/V	1.362 (0.472, 3.882)	0.569
Serum Parathyroid hormone (pmol/L)	0.994 (0.990, 1.013)	0.882
Serum total cholesterol (mmol/L)	0.812 (0.583, 1.142)	0.233
Serum triglycerides (mmol/L)	0.773 (0.594, 0.993)	0.044

DM: diabetes mellitus; rHuEPO: recombinant human erythropoietin; IGF-1: insulin-like growth factor-1; Kt/v: urea clearance index used to quantify the dialysis treatment adequacy, where K is the dialyzer clearance of urea, t is the dialysis time, and V is the volume of distribution of urea approximately equal to the total body water of the patient.

**Table 4.** Results of multiple linear regression models relating indicators of insulin growth factor-1 (independent variables) with hemoglobin (dependent variable).

	Model 1	Model 2	Model 3	Model 4	Model 5	Model 6	Model 7	Model 8	Model 9
IGF-1(ng/mL)	0.04	0.04	0.04	0.04	0.04	0.04	0.04	0.03	0.04
	(0.01–0.07)	(0.01–0.07)	(0.01–0.08)	(0–0.08)	(0–0.07)	(0–0.07)	(0–0.07)	(–0.01–0.07)	(0–0.08)
*p* value	0.007	0.009	0.02	0.032	0.052	0.051	0.051	0.098	0.061
IGF-1 < 197.03 ng/mL	0(Ref)	0(Ref)	0(Ref)	0(Ref)	0(Ref)	0(Ref)	0(Ref)	0(Ref)	0(Ref)
IGF-1 ≥ 197.03 ng/mL	8.02	7.92	8.17	7.69	7.05	6.93	7.18	6.47	7.07
	(2.99–13.05)	(2.88–12.96)	(2.66–13.68)	(2.05–13.34)	(1.33–12.77)	(1.07–12.79)	(1.52–12.83)	(0.8–12.13)	(1.17–12.97)
*p* value	0.002	0.002	0.004	0.008	0.017	0.022	0.014	0.027	0.020

Model 1: Unadjusted.

Model 2: Adjusted for DM.

Model 3: Adjusted for age, sex, dialysis vintage, and DM.

Model 4: Adjusted for age, sex, dialysis vintage, DM, and TG.

Model 5: Adjusted for age, sex, dialysis vintage, DM, TG, and hsCRP.

Model 6: Adjusted for age, sex, dialysis vintage, DM, TG, hsCRP, and rHuEPO dosage.

Model 7: Adjusted for age, sex, dialysis vintage, DM, TG, hsCRP, and iron supplementation.

Model 8: Adjusted for age, sex, dialysis vintage, DM, TG, hsCRP, and albumin.

Model 9: Adjusted for age, sex, dialysis vintage, DM, TG, hsCRP, and prealbumin.

**Table 5.** Results of multivariate binary logistic regression analyses of anemia (dependent variable) according to related IGF-1 indicators (independent variables).

	Model 1	Model 2	Model 3	Model 4	Model 5	Model 6	Model 7	Model 8	Model 9
IGF-1(ng/mL)	0.99	0.99	0.99	0.99	0.99	0.99	0.97	0.99	0.99
	(0.99–1)	(0.99–1)	(0.99–1)	(0.99–1)	(0.99–1)	(0.99–1)	(0.99–1)	(0.99–1)	(0.99–1)
*p* value	0.01	0.012	0.011	0.012	0.018	0.014	0.016	0.018	0.011
IGF-1 < 197.03 ng/mL	1(Ref)	1(Ref)	1(Ref)	1(Ref)	1(Ref)	1(Ref)	1(Ref)	1(Ref)	1(Ref)
IGF-1 ≥ 197.03 ng/mL	0.36	0.36	0.31	0.3	0.32	0.31	0.31	0.32	0.29
	(0.17–0.75)	(0.17–0.76	(0.13–0.72)	(0.13–0.72)	(0.13–0.78)	(0.13–0.76)	(0.13–0.75)	(0.13–0.78)	(0.12–0.74)
*p* value	0.007	0.007	0.006	0.007	0.012	0.01	0.009	0.012	0.009

Model 1: Unadjusted.

Model 2: Adjusted for DM.

Model 3: Adjusted for age, sex, dialysis vintage, and DM.

Model 4: Adjusted for age, sex, dialysis vintage, DM, and TG.

Model 5: Adjusted for age, sex, dialysis vintage, DM, TG, and hsCRP.

Model 6: Adjusted for age, sex, dialysis vintage, DM, TG, hsCRP and rHuEPO dosage.

Model 7: Adjusted for age, sex, dialysis vintage, DM, TG, hsCRP, and iron supplementation.

Model 8: Adjusted for age, sex, dialysis vintage, DM, TG, hsCRP, and albumin.

Model 9: Adjusted for age, sex, dialysis vintage, DM, TG, hsCRP, and prealbumin.

## Discussion

Our study showed that after adjusting for confounding factors, a low serum IGF-1 level was independently associated with anemia in patients undergoing MHD in different multivariable regression models, and positive correlations were observed between IGF-1 levels and albumin, prealbumin, and transferrin levels in this population.

As an active protein polypeptide, IGF-1 is produced by various cells, including those of the liver, kidney, and spleen. Many different cell types in the body express IGF-1 receptors. IGF-1 is synthesized in various tissues and acts in an autocrine or paracrine manner to promote cell growth or division. Studies have shown that patients with chronic kidney disease (CKD) and anemia have lower serum IGF-1 levels [9].

However, the underlying factors associated with these findings are complex. First, IGF-1 promotes erythropoiesis both *in vitro* and *in vivo* [10–12] and stimulates the proliferation and differentiation of late primitive erythroid progenitors and/or early erythroid progenitors, although the specific mechanism is not clear. Shimon et al. [13] found that the specific knockdown of megakaryocyte and platelet C-type lectin-like receptor 2 (CLEC-2) decreased IGF-1 concentrations in the serum and extracellular fluid of bone marrow, with an increase in apoptosis of bone marrow erythroid cells, which increased the risk of anemia. In this study, pretreatment with IGF-1 receptor inhibitors increased the rate of apoptosis and reduced erythroblast proliferation *in vitro*. Therefore, the authors suggest that IGF-1 secretion from podoplanin (PDPN)-expressing stromal cells by CLEC-2 stimulation positively regulates erythroblasts.

Second, IGF-1 regulates several physiological processes, including cell proliferation and migration, cell growth, angiogenesis, and apoptosis, by activating the mitogen-activated protein kinase (MAPK) and phosphatidylinositol 3-kinase/Akt signaling pathways [14]. Terrence et al. [15] reported that mesenchymal stromal cells that were genetically engineered to overexpress IGF-1 enhanced the effects of cell-based gene therapy for renal failure-induced anemia treated with IGF-1 and that IGF-1-mediated proliferation might play a role in this process. Third, IGF-1 synergistically induced the expression of Bcl-xL in the BaF3 pro-B-cell line, which is essential for the inhibition of apoptosis and promotion of erythropoiesis [16]. Additionally, IGF-1 reduces the expression of inflammatory molecules and decreases the levels of C-reactive protein and fibrinogen [17]. Moreover, inflammatory status is an important factor for anemia in patients undergoing MHD [18]. A decrease in circulating IGF-1 may lead to an increase in the concentration of inflammatory proteins, which may affect erythropoietin secretion and erythropoiesis [19]. Furthermore, it has been suggested that decreased IGF-1 levels may be responsible for reduced erythropoietin activity and iron metabolism [20]. It can be inferred that IGF-1 may exert a proerythropoietic effect through a variety of potential mechanisms.

In patients newly requiring hemodialysis, low serum IGF-1 levels are correlated with body composition as well as mineral and bone metabolism markers and predict an increased risk of mortality. Serum IGF-1 is a nutritional status marker in patients with end-stage renal disease (ESRD) and is negatively correlated with the subjective global assessment (SGA) nutritional status in patients undergoing hemodialysis [21]. Our findings demonstrate a positive correlation between serum albumin, prealbumin, and IGF-1 levels, indicating that the impact of IGF-1 on nutrition may also play a role in anemia. However, the specific mechanisms underlying this association require further investigation.

Intervention studies have shown that in malnourished patients undergoing dialysis [22] and older adults [23], short-term treatment with recombinant human growth hormone (GH) could increase hemoglobin levels and thus improve anemia. By regulating the amount of IGF-1 synthesized and released by the liver or by modulating the plasma concentrations of IGF-1-binding proteins, GH can affect the amount of free IGF-1 bound to receptors on the cell surface, which reflects the bioavailability of IGF-1. Disturbances in GH and IGF-1 bioavailability may lead to renal anemia [24]. Kim et al. found that the levels of free IGF-1 and its bioactivity in patients undergoing MHD were approximately half of those observed in healthy participants. During dialysis, the bioactivity of IGF-1 decreases by >50%. Insulin-like growth factor-binding proteins (IGFBPs) are important regulators of IGF-1 action. Reduced renal clearance leads to the accumulation of intact and partially degraded IGFBPs in serum, resulting in the downregulation of free IGF-1 levels and activity [25]. In summary, IGF-1 may be an important factor in the regulation of erythropoiesis in patients with CKD. Although during our literature search, we found that most studies on the relationship between serum IGF-1 and hemoglobin in patients with CKD or on dialysis were published in the late 20th and early 21st centuries, no study has yet to show that exogenous IGF-1 preparations can improve anemia in this population.

This study has several limitations. First, it had a cross-sectional design; thus, a causal relationship between serum IGF-1 levels and anemia could not be determined. Second, this was a single-center study with a small sample size; therefore, it had limited statistical power and possible unknown biases. Third, the researchers measured hemoglobin and IGF-1 only once and did not perform repeated measurements at different time points; therefore, the association between IGF-1 and hemoglobin levels needs to be further confirmed.

## Conclusion

In summary, we found that low serum IGF-1 levels were independently associated with anemia and lower hemoglobin levels in patients undergoing MHD. Thus, the erythropoietic function of IGF-1 may be an associated factor. However, prospective studies with large sample sizes and multiple centers need to be conducted. Furthermore, serum IGF-1 and hemoglobin levels should be repeatedly measured to confirm the associations between the two to provide a theoretical basis for prospective and interventional studies on the treatment of IGF-1 in patients undergoing MHD with anemia.

## Data Availability

The datasets used and/or analyzed during the current study are available from the corresponding author upon reasonable request.
